# Clinical results of XMR-assisted percutaneous transforaminal endoscopic lumbar discectomy

**DOI:** 10.1186/1749-799X-8-14

**Published:** 2013-05-25

**Authors:** Gun Choi, Hitesh N Modi, Nicolas Prada, Tae-Joon Ahn, Sung Hee Myung, Mi Sun Gang, Sang-Ho Lee

**Affiliations:** 1 Department of Neurosurgery, Wooridul Spine Hospital, Seoul 135-100, South Korea; 2 Department of Orthopedic Surgery, Wooridul Spine Hospital, 47-4, Chungdam-dong, Gangnam-gu, Seoul 135-100, South Korea; 3 Department of Nursing, Wooridul Spine Hospital, Seoul 135-100, South Korea

**Keywords:** Percutaneous endoscopic lumbar discectomy, Incomplete disc removal, XMR-guided procedure, High success rate

## Abstract

**Background:**

Although percutaneous endoscopic lumbar discectomy (PELD) has shown favorable outcomes in the majority of lumbar discectomy cases, there were also some failures. The most common cause of failure is the incomplete removal of disc fragments. The skin entry point for the guide-needle trajectory and the optimal placement of the working sleeve are largely blind, which might lead to the inadequate removal of disc fragments. The objective of this study was to present our early experiences with image-guided PELD using a specially designed fluoroscope with magnetic resonance imaging-equipped operative suite (XMR) for the treatment of lumbar disc herniation.

**Methods:**

This prospective study included 89 patients who had undergone PELD via the transforaminal approach using an XMR protocol. Pre- and postoperative examinations (at 12 weeks) included a detailed clinical history, visual analogue scale (VAS), Oswestry disability index (ODI), and radiological workups. The results were categorized as excellent, good, fair, and poor according to MacNab's criteria. At the final follow-up, the minimum follow-up time for the subjects was 2 years. The need for revision surgeries and postoperative complications were noted on follow-up.

**Results:**

Postoperative mean ODI decreased from 67.4% to 5.61%. Mean VAS score for back and leg pain improved significantly from 4 to 2.3 and from 7.99 to 1.04, respectively. Four (4.49%) patients underwent a second-stage PELD after intraoperative XMR had shown remnant fragments after the first stage. As per MacNab's criteria, 76 patients (85.4%) showed excellent, 8 (8.89%) good, 3 (3.37%) fair, and 2 (2.25) poor results. Four (4.49%) patients had remnant disc fragments on XMR, which were removed during the same procedure. All of these patients had either highly migrated or sequestrated disc fragments preoperatively. Four (4.49%) other patients needed a second, open surgery due to symptomatic postoperative hematoma (*n* = 2) and recurrent disc herniation (*n* = 2).

**Conclusions:**

This prospective analysis indicates that XMR-assisted PELD provides a precise skin entry point. It also confirms that decompression occurs intraoperatively, which negates the need for a separate surgery and thus increases the success rate of PELD, particularly in highly migrated or sequestrated discs. However, further extensive experience is required to confirm the advantages and feasibility of PELD in terms of cost effectiveness.

## Introduction

Traditional open lumbar laminectomy and discectomy were modified to a more recent microlumbar discectomy technique, which is a less-invasive procedure for the treatment of lumbar disc herniation. However, this method has potential disadvantages, such as paraspinal muscle damage, iatrogenic instability, epidural fibrosis, a higher probability of retraction injuries to neural tissue, and disc space collapse leading to facet arthropathy [1]–[5]. With recent developments in the field of minimally invasive spine surgery and with the aim of preserving as much normal spinal anatomy as possible, percutaneous endoscopic lumbar discectomy (PELD) has emerged as one of the most favored minimally invasive procedures for lumbar disc herniation [6]. PELD, through the transforaminal posterolateral approach under local anesthesia, as described by Kambin et al. [7], was classically devised for contained soft disc herniation. Hijikata [8] performed the first percutaneous nucleotomy procedure in 1975, and Kambin and Gellman reported their first experience with the method in 1983 [7]. Since then, PELD has gradually emerged as the treatment of choice for a variety of herniated discs. With recent inclusions of migrated or even sequestrated fragments as indications [9,10], it is becoming a truly comprehensive treatment of disc ruptures [11]–[17]. The major advantage of PELD, in addition to being minimally invasive [18,19], is that surgery is done under local anesthesia, promoting rapid recovery and less morbidity. The importance of advanced radiological imaging, improved optics, and improved instruments makes PELD more versatile than other techniques.

With the increasing interest in PELD, some of its limitations have been discussed in the literature. A major concern with PELD is the risk that revision surgery will be required due to the possibility of remnant disc fragments postoperatively [20] and also due to the difficulty in deciding on a proper and safe skin entry point, using a guide needle [21]. The possibility of inadequate decompression is even greater with high-grade migration (HGM) or high-grade canal compromise (HCC) [20]. To overcome these pitfalls, we report our early experience with a new image-guided PELD, using a specially designed fluoroscope (Allura 15″, Phillips Medical System, Veenpluis, Netherlands; routinely used in cardiac cath labs) with a magnetic resonance imaging (MRI; Achieva 1.5T, Philips Medical Systems, Veenpluis, Netherlands)-equipped operative suite (XMR). The integration of a fluoroscope with an MR is now being used both enthusiastically and successfully in interventional cardiology. To our knowledge, this is the first installation of this integration for spinal navigation surgery. The aim of this article was to present the clinical and radiological results of XMR-guided PELD for lumbar disc herniation in the 89 cases included in our patient series.

## Materials and methods

We received an Ethical Committee (Seoul Wooridul Hospital IRB) approval for this study prior to commencing it. We prospectively evaluated 89 consecutive patients who underwent XMR-assisted PELD. The subjects included 30 women and 59 men with an average age of 46.6 years. The inclusion criteria were as follows: (1) lower limb radiculopathy; (2) presence of root tension signs (sciatic or femoral nerve); (3) failure of adequate (6 weeks) and supervised conservative treatment with lifestyle modifications, nerve root/epidural blocks, nonsteroidal antiinflammatory drugs, and physiotherapy (or early if the pain was severe or the patient showed neurological deterioration); and (4) corroborative clinical and radiological findings. Patients with (1) cauda equina syndrome, (2) severe central canal stenosis, and (3) associated segmental instability were excluded from the study.

**Figure 1:**
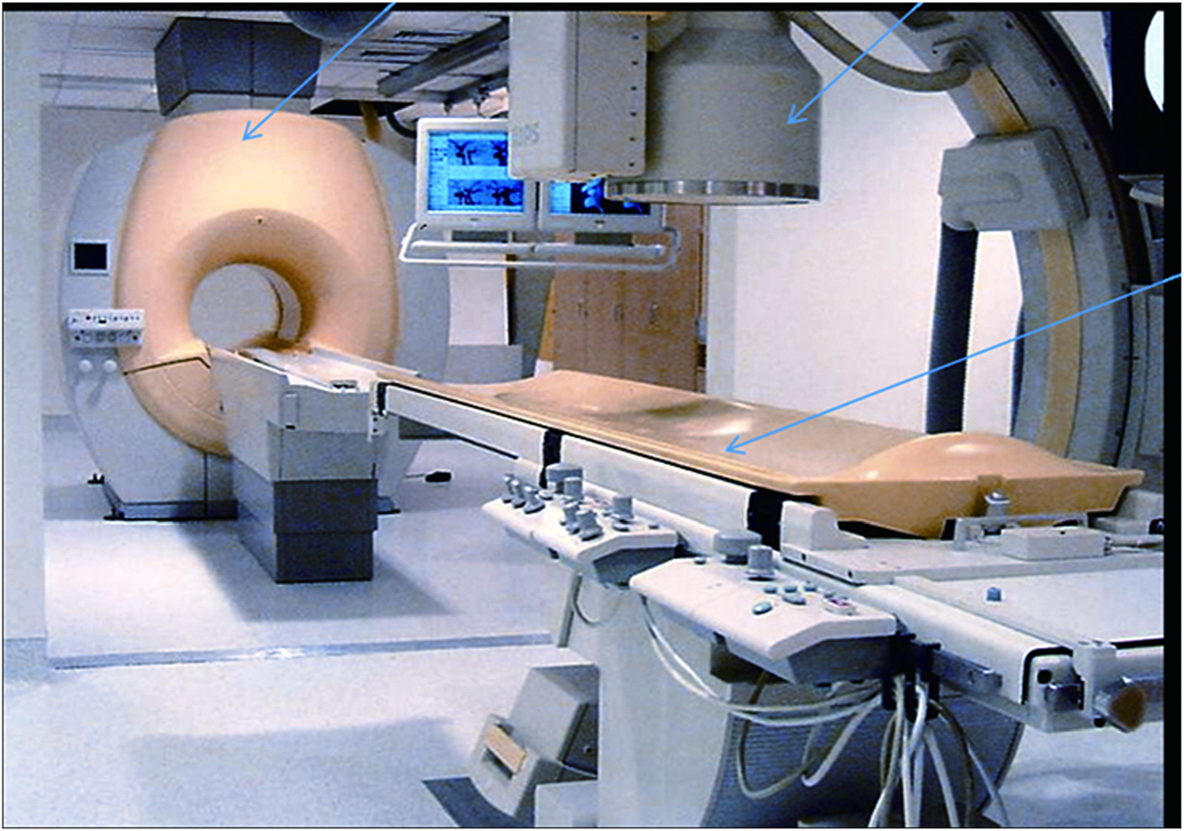
**XMR suite: arrows indicating (from left to right) the MRI, fluoroscope, and sliding table.** The glass door between the MRI and the sliding table can also be seen.

**Figure 2:**
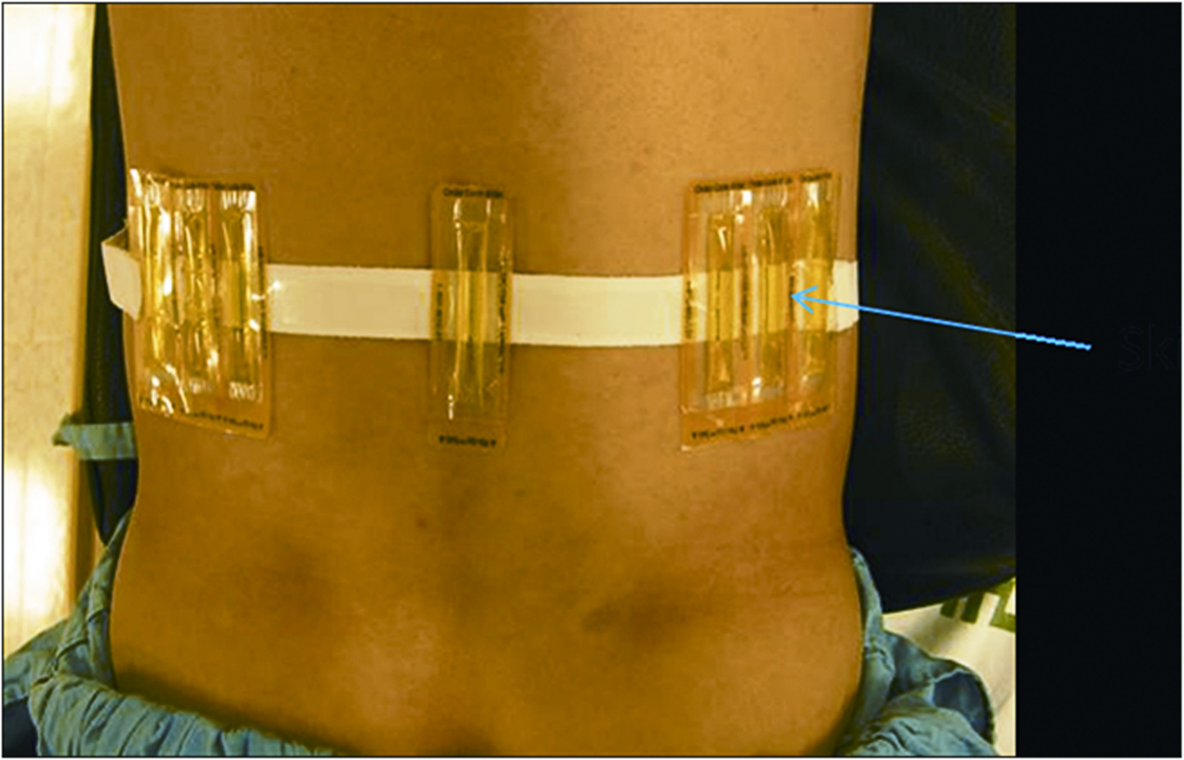
**Patient with attached skin markers (MR-SPOTS, Beekley Corp, Bristol, CT, USA).** Based on the markers, the exact trajectory for the entry guide pin can be decided.

**Figure 3:**
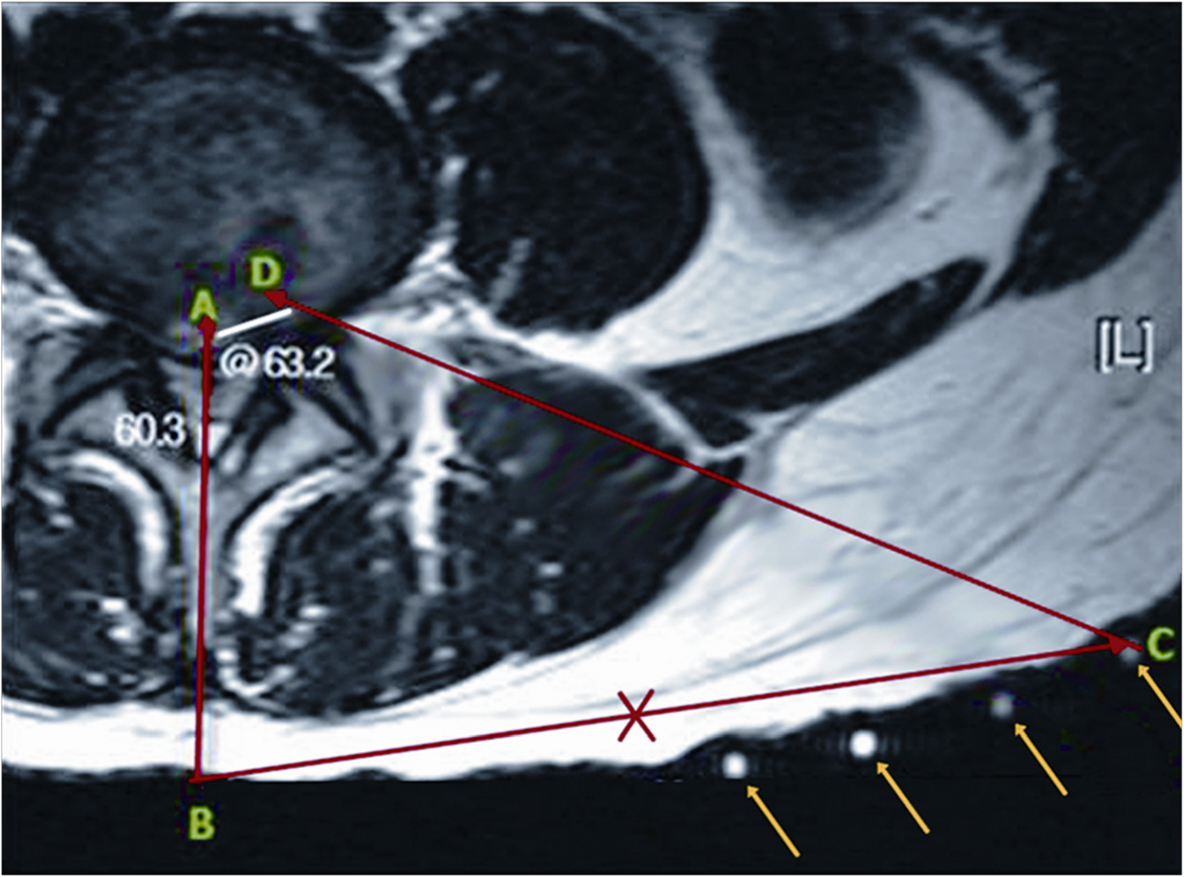
**Preoperative planning for XMR-guided skin entry point and subsequent needle trajectory (all skin markers - arrows).** Different markers at position **C** are placed during XMR, and one of these markers was selected as the correct entry point, as shown in the picture. Line **AB** is the midline; **CD** is the proposed entry point to reach the target; and **BC** is the distance of the skin entry point from the midline.

All patients were evaluated preoperatively by detailed clinical history, physical examination, visual analogue scale (VAS) for back and leg pain, Oswestry disability index (ODI), and radiological imaging (radiographs of the lumbosacral spine, including dynamic views, MR, and CT scan). We have installed a new system that integrates X-ray and MR imaging (an XMR suite) with a table (an Achieva I/T Cardiovascular or XMR combining an Achieva 1.5T CV and an X-ray system; Philips Medical System) that can slide from the MR to the fluoroscope suite and vice versa. This permits rapid, smooth, intermodality patient transport by trained operation theater staff (Figure 1). The XMR suite consists of independently operating MRI and fluoroscope separated by radio frequency-shielded sliding doors. In-lab images are displayed on LCD screens. The patient can be shifted from one modality to another on the sliding table without shifting or changing the surgical position. The patients were allowed to choose if they wanted to undergo the XMR protocol after thorough discussions and explanations about the procedure. The XMR protocol demands immediate preoperative MR (T2-weighted sagittal and axial MR) with attached skin markers (MR-SPOTS, Beekley Corp, Bristol, CT, USA) (Figure 2) in the XMR suite, one MR scan during the procedure to check the decompression and a further scan if a remnant fragment is seen on intraoperative MR (T2 weighted) after primary removal is contemplated. After the prior consent, each patient was subjected to a preoperative MR (T2 images) scan in the XMR suite. For the procedure, they were placed in the operative prone position on the sliding table with attached skin entry point markers just before the surgical procedure. The entry point was decided based on the MR markers (Figure 3), keeping in mind: (1) the safety of the needle track (avoiding vessels and the retroperitoneal space and neural structures visualized on MR) and (2) the ease of reaching the pathology. The entry point was selected from the different points of the MR markers on the skin. After confirmation and marking of the level of the disc herniation, the patient was moved from the MR to the fluoroscopic suite. Patients underwent PELD using the Yeung endoscopic spine system (Richard Wolf, Knittlingen, Germany) with the same standard protocols and surgical steps through a transforaminal approach under local anesthesia.

**Figure 4:**
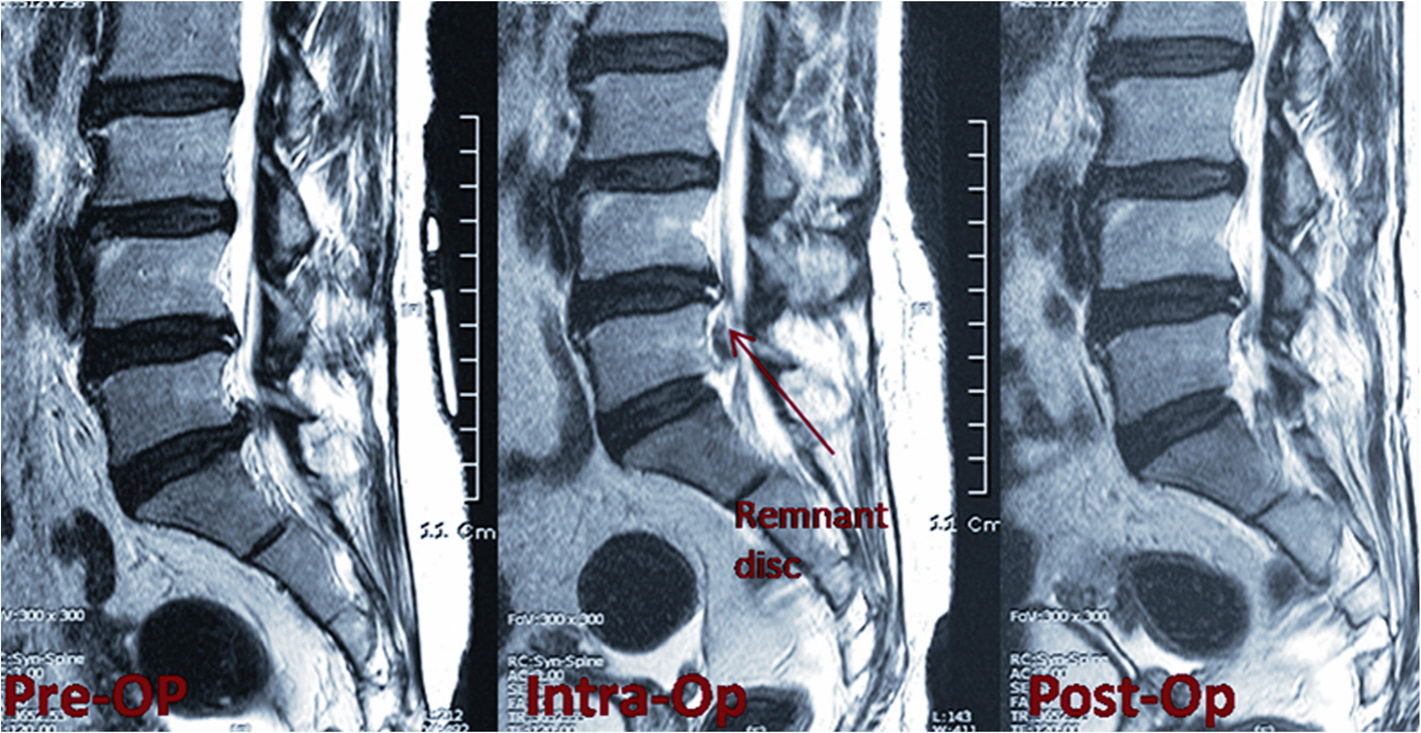
Intraoperative XMR confirming the remnant fragments and final MRI showing complete decompression.

Once the PELD procedure was finished, the scope was removed, and a sterile plastic tube was placed with a sterile adhesive over the wound to avoid a new entry point for the guide wire if further surgery was planned. The patient (without changing the operating position) was shifted on the operating table back to the MR for an intraoperative MR scan to check the adequacy of the decompression. If any remnant fragments were found at this stage (Figure 4), the patient was again shifted to the fluoroscopy suite in the same position on the table for the removal of the remnant fragment. Standard postoperative regimens were prescribed for all patients, who were discharged the following day, prescribed with prophylactic oral antibiotics for 6 days.

All patients were followed up at 12 weeks by telephonic interview, mailed questionnaire, or hospital follow-up. The postoperative VAS score and ODI were charted. The results were categorized as excellent, good, fair, and poor based on MacNab's criteria. MacNab's criteria were measured as follows: ‘excellent’ is a state without pain, without restriction of movement, and would allow the patient to function normally; ‘good’ is a state with occasional pain but which would allow the patient to function normally; ‘fair’ indicates slight progress; and ‘poor’ reflects no progress. The same evaluation was done at the final follow-up, with a minimum follow-up of 2 years.

## Results

The mean duration for back and leg pain was 156.8 and 18 weeks, respectively. Eight, 19, 49, and 13 patients had disc herniation at the L2-3, L3-4, L4-5, and L5-S1 levels, respectively, on MRI, which correlated with clinical findings. Out of 89 patients who underwent preoperative XMR as part of the protocol, seven showed a change in disc size and/or location, as compared to previous imaging studies. The mean operative time for transforaminal PELD was 60 min.

The postoperative mean ODI decreased from 67.4% (preoperative) to 5.61% (postoperative), and the mean VAS score for back and leg pain improved significantly from 4 to 2.3 and from 7.99 to 1.04, respectively. Four (4.49%) patients underwent a second-stage PELD after intraoperative XMR due to remnant fragments after the first stage. All of these fragments were diagnosed preoperatively either as a highly migrated or sequestrated disc. As per MacNab's criteria, 76 patients (85.4%) showed excellent, 8 (8.89%) good, 3 (3.37%) fair, and 2 (2.25%) poor results. Four (4.49%) more patients required a separate, second surgery due to persistent symptoms even after complete fragment removal had been confirmed using XMR (*n* = 2) and reappearance of symptoms during the first 2 weeks after the primary procedure (*n* = 2). These four patients underwent a separate open surgery; two had a symptomatic hematoma and the other two had true recurrent disc herniation within 2 weeks of the first surgery. Besides these cases, there were no other intraoperative or postoperative complications or infections up until the latest follow-up. Furthermore, the patients' VAS for back and leg pain and ODI were maintained at the final follow-up compared with postoperative scores.

## Discussion

Since the discovery of X-rays in 1895, advances in medical imaging have revolutionized not only the process of diagnosis but also of therapy. Using image guidance, surgical procedures can be replaced by less-invasive alternatives with higher accuracy. Minimally invasive surgery has recently revolutionized all fields of surgery. Kambin introduced the concept of posterolateral percutaneous lumbar disc decompression in 1973 [19]. During its early days, the focus in percutaneous procedures was limited to manual or automated percutaneous decompression, relying more on central debulking with the reduction of intradiscal pressure. With the growing experience and advances in medical technology, an increasing number of disc herniation patterns have become accessible through endoscopy. Some of the previous difficult indications such as high migration, foraminal or extraforaminal herniation, and sequestrated discs are now being easily dealt with by PELD [13,14,22].

Since the inception of PELD, two major issues have been identified as critical for surgeons. One is the possibility of remnant fragments or inadequate decompression of neural tissue, and the other is how to decide on an exact skin entry point for the guide wire that will assure a successful PELD [20]. It is recognized that these drawbacks are connected with each other; thus, if the skin entry point is based on accurate measurements, the probability of completely removing the herniated disc fragment is increased. As reported in previous studies [7,9,10,15,16,19,20], identifying a precise skin entry point, using a needle that guides the position of subsequent instruments inside the disc space, is the most crucial step for a successful PELD. A herniated fragment is accessible only when the surgical instruments are placed in the optimal trajectory [21]. Routinely, the skin entry point is selected based on experience, about 12 ± 2 cm lateral from the midline as described by Yeung et al. [9,10]. The target is the medial-pedicle line in the AP view and posterior vertebral line in the lateral view to reach Kambin's triangle, traversing the postulated safe trajectory [21]. With the growing experience in PELD, it has been noted that an incorrect entry point poses the risk of damaging vital structures such as intraspinal neural elements and small retroperitoneal vessels, resulting in retroperitoneal hematomas that require urgent drainage. With XMR labeling of the skin entry point, which is done in the same prone position as surgery and on the same operation table without changing the position of patient, we were able to decide on the most effective entry point. This allowed us to address the pathology without causing injury to any vital structures, through a safe trajectory, and to achieve complete removal of the herniated fragments. To our knowledge, this is the first article reporting the outcome of the PELD procedure for lumbar disc herniation using XMR.

As reported by Lee et al. [20], the main consequence of PELD failure is reoperation, mainly due to inadequate decompression resulting in persistent radiculopathy. They concluded that the rate of operative failure due to inadequate decompression differed significantly according to the size and location of the herniation. In nonmigrated herniation, central HCC (>50% canal compromise) herniation showed the highest rate of failure (15%), and the failure rate was significantly different between the low-canal compromise and HCC groups (1.9% and 11.1%, respectively; *P* < 0.001). There was no significant difference in the failure rate between the nonmigrated herniation and low-grade migration groups (2.7% and 3.7%, respectively). However, the HGM group (migration upward or downward beyond the measured height of the posterior marginal disc space) showed a significantly high incidence of failure (15.7%, *P* < 0.001). Based on these results, Lee et al. concluded that open surgery may be considered for herniation with HCC and/or HGM. Schaffer and Kambin [23] analyzed 11 patients who had undergone reoperation out of 100 patients who had been treated with PELD. Of these, five had persistent symptoms after endoscopic surgery. The most common reasons for subsequent surgery were lateral recess stenosis, remnant herniation, and improper placement of the working instrument. The greatest disadvantage of a second surgery was patient dissatisfaction, which could lead to disturbed doctor-patient relations. XMR reduces the possibility of a second surgery, as it makes the system more useful for patients with HGM and HCC. Before moving the patient out of the surgical suit, XMR diagnoses these inadequate decompressions (Figure 4). With our especially designed XMR suite, we were able to deal with remnant fragments (which might have otherwise resulted in a separate second surgery) without shifting or even changing the position of the patient on the operating table. This made the second stage (i.e., the second stage of the same surgery and not a separate second surgery) a smooth transition from the first part of the surgery, thus avoiding patient dissatisfaction. XMR helped us achieve a good success rate in removing the compressing disc fragments without causing patient dissatisfaction. However, if the herniation reoccurs after successful removal of the disc, the possibility of requiring further surgery cannot be completely ruled out. In our case series, we successfully removed remnants of disc fragments using XMR in four patients who had HGM; however, the other four patients had to undergo revision surgery mainly due to postoperative hematoma or recurrent disc herniation.

The distribution of patients with HCC and HGM in our series was 22.47% and 21.35%, respectively, which was comparatively higher than that in Lee et al. [20] (6.24% and 4.41% cases, respectively). Therefore, it was likely that there would be remnant fragments after the surgery. In our cases, the remnant fragments were either highly migrated or sequestrated disc fragments. Despite having such a large number of patients with HCC and HGM discs, we were able to remove the herniated fragments almost completely (probably because of the accuracy of the XMR-assisted needle placement and the intraoperative identification of remnant fragments). In addition, we avoided open surgeries in these patients. In fact, according to the recommendations by Lee et al., these patients are candidates for open surgery. Only 4.49% (4/89) needed a second-stage surgery (according to the XMR protocol; the second stage is commenced after intraoperative XMR identification of remnant fragments) despite such a large distribution of difficult disc herniations. Our main reason for selecting a very small number of patients for the second stage of the operation was that the XMR guided us in tracking the correct trajectory for the entry point, which if incorrect is one of the major causes of failure.

**Figure 5:**
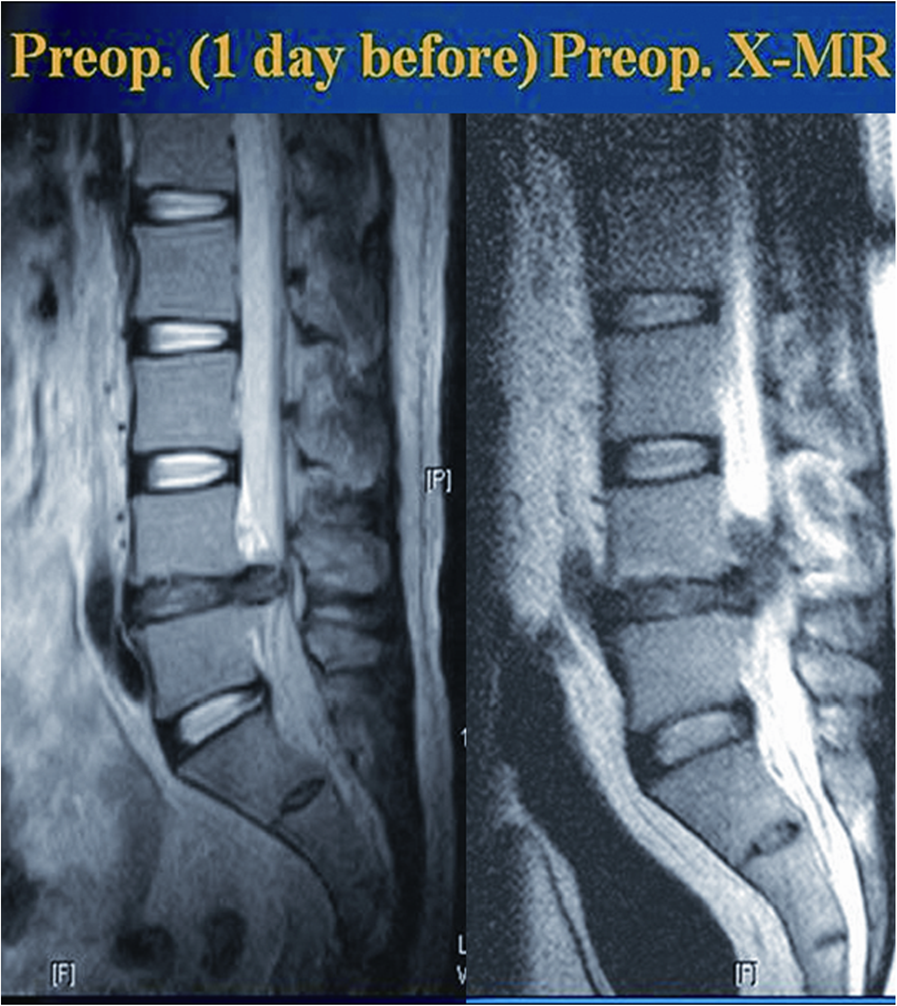
**Preoperative XMR confirming the change in location and size of the herniated fragment.** Postoperative MR showing a decompressed fragment.

Our experience taught us that the herniated disc might change in size and/or location during the time interval between preoperative MRI and the actual surgery (Figure 5). To achieve a successful decompression with PELD, the surgeon must be aware of the exact location and extent of the disc herniation. XMR just before surgery avoids any error in the surgeon's assessment of the herniated disc fragment inside the spinal canal, leading to a satisfactory outcome. We agree that a change in the disc fragment position is associated with a change in the severity of clinical symptoms. However, this can be checked on a second conventional MR. We like to emphasize that our preoperative MR is used not only to diagnose this change in position but also to mark the precise skin entry point. Preoperative fragment orientation on XMR (Figure 5) is merely an additional advantage that can eliminate the need for a separate conventional MR.

**Figure 6:**
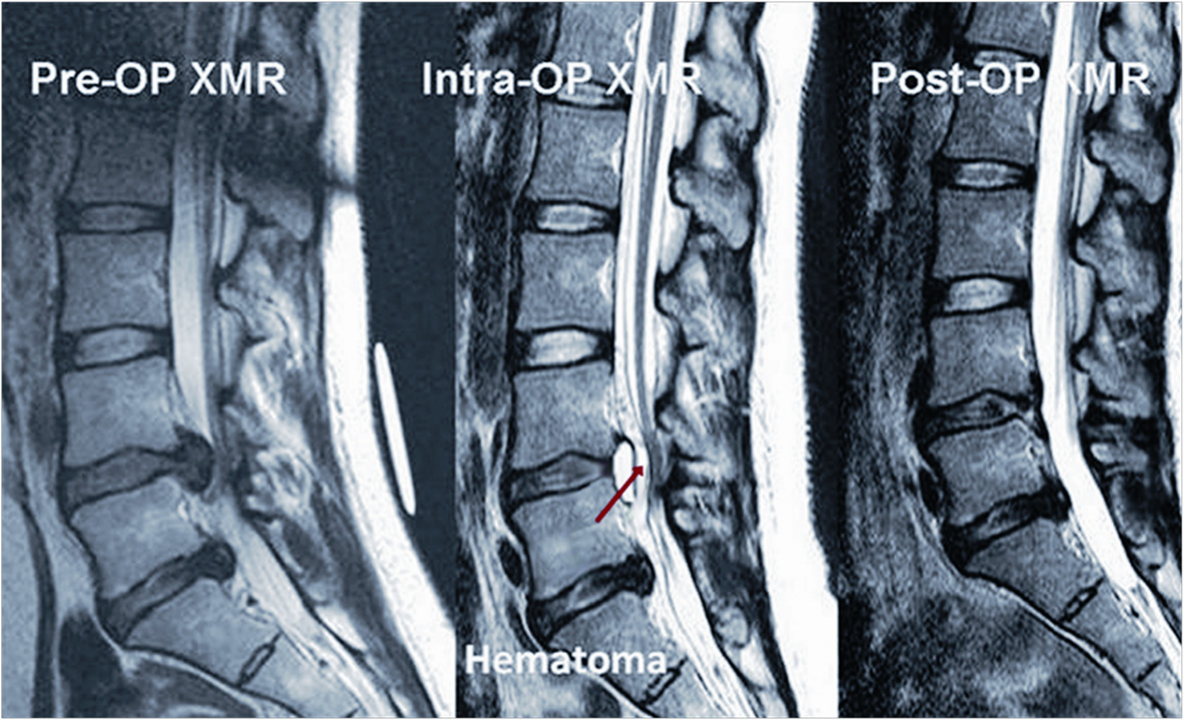
Intraoperative XMR indicating a hematoma, which was symptomatic and treated with percutaneous drainage through the transforaminal approach.

In the present study, four (4.49%) patients underwent a second subsequent open surgery despite confirmed decompression on postoperative XMR. Out of the four patients, two had persistent symptoms after PELD due to postoperative hematoma (Figure 6), and the other two came back with true recurrent disc herniation at the index level within 2 weeks of PELD. We believe that these complications are not different from those for the usual PELD or microscopic surgeries. However, XMR-assisted PELD definitely avoided revision surgeries in four patients who had true remnant fragments after the operation. We think that this is one of the advantages of using XMR, especially in highly migrated or sequestrated discs.

However, there are certain disadvantages of the XMR system. It increases the cost of the procedure, the inventories are expensive, and it requires trained and supportive staff. Additionally, there is of course a slight increase in the time a patient has to stay in the operating room. However, although the cost of the surgery is a little higher, the XMR system definitely avoids a high dissatisfaction rate after the surgery, especially in the cases of HCC and HGM discs. Moreover, nowadays, we are selectively using XMR-assisted PELD only for patients with highly migrated or sequestrated discs. Of course, patients should be given the choice after informing them of the advantages and disadvantages of the procedures, including the extra expense. However, from our experience, most patients choose XMR, showing that satisfaction and complete removal are more important factors for them than the cost of the procedure. Although there is no absolute contraindication of this technique for canal stenosis or other types of disc herniation, we emphasized using this technique in HCC and HGM discs not to miss the remnant disc fragments and to optimize the cost effectiveness of the procedure. Another factor is the use of antibiotics in our study. As this was our first experience with the XMR, which takes an extra 20 to 30 min to shift the patient to the MR suite, we followed the protocol by prescribing 6 days of extra oral antibiotics as a precautionary measure. However, we agree that this is not required, and we now give only one shot of preoperative intravenous antibiotics.

## Conclusions

The present prospective analysis indicates that XMR-assisted PELD can be performed with a precise skin entry point. Furthermore, it confirms decompression intraoperatively, thereby reducing the need for a separate, second surgery and increasing the success rate of PELD. However, it should be used in selected cases with high-grade migrated or sequestrated disc herniations.

## Competing interests

The authors declare that they no competing interests.

## Authors' contributions

Authors GC, SHL, and HNM participated in the study design and approving the work; NP, TJA, SHM, and MSG participated in the data collection; HNM and NP participated in the manuscript writing and data analysis; and GC, SHL, and TJA participated in the final revision and approval. All authors read and approved the final manuscript.
